# Induction of Labor with Vaginal Dinoprostone (PGE_2_) in Patients with a Previous Cesarean Section: Obstetric and Neonatal Outcomes

**DOI:** 10.3390/jcm10225221

**Published:** 2021-11-09

**Authors:** Nuria López-Jiménez, Fiamma García-Sánchez, Rafael Hernández Pailos, Valentin Rodrigo-Álvaro, Ana Pascual-Pedreño, María Moreno-Cid, Antonio Hernández-Martínez, Milagros Molina-Alarcón

**Affiliations:** 1 Department of Obstetrics and Gynecology, La Mancha Centro Hospital, 13600 Alcázar de San Juan, Spain; nurialj92@gmail.com (N.L.-J.); fiammagss@hotmail.com (F.G.-S.); rhdezalcalapailos@yahoo.es (R.H.P.); biovalen.r@gmail.com (V.R.-Á.); ginemancha@hotmail.com (A.P.-P.); mmorenocid@gmail.com (M.M.-C.); 2 Department of Nursing, Physiotherapy and Occupational Therapy, Faculty of Nursing, University of Castilla-La Man cha IDINE, Camilo José Cela, 14, 13071 Ciudad Real, Spain; Milagros.Molina@uclm.es

**Keywords:** induction of labor, trial of labor after cesarean (TOLAC), PGE_2_

## Abstract

Background: Vaginal dinoprostone (PGE_2_) is currently used as the prostaglandin of choice in many obstetric units. However, few studies have evaluated its safety, especially in women who previously had a cesarean section. Objective: To evaluate the efficacy and safety of PGE_2_ in pregnant women who are undergoing induction of labor (IOL), and who have had a previous cesarean section. Materials and Methods: A prospective observational study was conducted in La Mancha Centro Hospital in Alcázar de San Juan, Spain, from 1 February 2019 to 30 August 2020. Obstetric and neonatal outcomes, following IOL with PGE_2_, in 47 pregnant women who wanted a trial of labor after cesarean (TOLAC), and 377 pregnant women without a history of cesarean section, were analyzed. The outcomes were analyzed by bivariate and multivariate analyses using binary and multiple linear regression. Results: A total of 424 women were included in this study. The percentage of cesarean sections in the TOLAC group was 44.7% (21), compared with 31.6% (119) in the group without a history of cesarean section (adjusted odds ratio: 1.4; 95% CI: 0.68–2.86). In the multivariate analysis, no statistically significant differences were observed between both groups for obstetric and neonatal outcomes (*p* > 0.05). However, two uterine ruptures (4.3%) occurred in the group of patients with a history of cesarean section who underwent IOL with PGE_2_. Conclusions: The induction of labor with vaginal dinoprostone (PGE_2_), in patients with a previous history of cesarean section, was not associated with worse obstetric or neonatal outcomes compared with the group of patients without a history of cesarean section in our study sample. However, further research is needed regarding this IOL method, and it should be used with caution in this population group.

## 1. Introduction

Induction of labor (IOL) is an obstetric procedure that is conducted with increasing frequency worldwide, reaching a percentage of 29.4% in some countries, such as the US [1]. IOL is indicated when there is an increased risk for the mother or the fetus to continue the pregnancy. In those pregnant women who have previously undergone a cesarean section, and who wish to trial vaginal delivery, IOL is also a valid option. However, this procedure should be carried out with particular caution in this group of women, due to the significantly increased risk of uterine rupture, compared with those that present with spontaneous induction of labor [2] (1.5% versus 0.8%). The incidence of rupture is also higher when compared to expectant management (1.3 to 1.4% vs. 0.4 to 0.6%) [3,4], although the absolute risk is low.

There is no current consensus regarding the most appropriate method for cervical ripening or IOL in this group of women [5]. The quality of evidence available is low, and there is a lack of sufficient information coming from randomized clinical trials comparing some methods with others [5]. Among the cervical ripening methods available, mechanical dilation with a balloon catheter is the method of choice recommended by some scientific societies, such as ACOG or SOGC, in patients with a previous cesarean section. This is due to the lower rate of tachysystole with changes in fetal heart rate (FHR) [6], as well as the lack of conclusive results suggesting an increased risk of uterine rupture compared to pharmacological dilation [7,8].

On the other hand, the use of prostaglandins for cervical ripening and IOL in patients with a previous cesarean section is controversial. Various studies have shown that the use of misoprostol (prostaglandin E1) is associated with a clear increase in the risk of uterine rupture and perinatal morbidity (RR: 3.67; 95% CI: 0.16–84.66) [9,10]; hence, some scientific societies contraindicate its use [7,8,11]. However, studies that have evaluated cervical ripening with prostaglandin E2 (PGE_2_) have shown conflicting results [12,13]. For this reason, in the absence of conclusive results, many countries continue to use PGE_2_ for IOL.

Therefore, with the aim of increasing the evidence in this field, our efforts have been directed at evaluating the efficacy and safety of IOL and cervical ripening with vaginal dinoprostone (PGE_2_), in patients with a previous cesarean section, comparing their obstetric and neonatal outcomes with those of women who are undergoing IOL without a history of cesarean section.

## 2. Materials and Methods

A prospective observational study was carried out from 1 February 2019 to 20 August 2020 at the La Mancha Centro Hospital in Alcázar de San Juan, Spain. This study has been approved by the center’s Clinical Research Ethics Committee (CREC), with protocol number 102-C. All the patients who participated in the study did so voluntarily and anonymously after signing informed consent.

The study population included a consecutive sample of singleton pregnancies in cephalic presentation with a history of previous cesarean section, desire to trial labor after cesarean (TOLAC), and with a medical indication for IOL and cervical ripening with PGE_2_, following the guidelines of the Spanish Society of Gynecology and Obstetrics [11]. There were no restrictions regarding gestational age and parity. Multiple gestation pregnancies, non-cephalic presentations, pregnancies with more than one previous cesarean section, antepartum fetal deaths, and patients who did not consent to participate in the study were excluded. The study group was compared with singleton pregnancies in cephalic presentation without a history of previous cesarean section, who also required IOL and cervical ripening with PGE_2_.

### 2.1. Information Sources

A specific database was created for data collection that included all study variables obtained from computerized medical records and partographs. In addition, any medical history not found in the medical files was obtained through personal interviews.

The main independent variable was the existence of a previous cesarean section (no/yes), while the dependent variables were obstetric and neonatal outcomes. Sociodemographic and obstetric variables were used as control variables (Table 1).

### 2.2. IOL Protocol at the Study Center

Women who wished to attempt a TOLAC were informed about the IOL and cervical ripening method used, as well as the risks and benefits of attempting vaginal delivery. The Spanish Society of Gynecology and Obstetrics (SEGO) guidelines were used for IOL indications [11]. In our center, in those patients who present an unfavorable cervix (Bishop ≤ 6), IOL begins with a cervical ripening process, involving administering a vaginal device that slowly releases 10 mg of PGE_2_ at a rate of 0.3 mg/h in 24 h (Propess^®^). Once the device has been placed, continuous cardiotocography (CTG) is performed on the patient for 2 h. If, after insertion, the fetal heart rate is classified as non-reassuring—NICHD II or III (according to the system proposed by the National Institute of Child Health and Human Development (NICHD) [14])—or uterine tachysystole is observed (>5 contractions in 10 min), the device is removed immediately. If no incidence occurs, a new CTG is performed at 12 and 24 h, and the device is removed when the patient presents a favorable cervix (Bishop > 6) or is at 3–4 cm dilation with regular uterine contractions. If after 24 h there has been no change in cervical conditions, the device is withdrawn, and induction is continued by intravenous oxytocin infusion and artificial rupture of the membranes using a standardized method if needed.

Specifically, oxytocin is administered intravenously via an infusion pump, at dose of 2 mU/min, with a time period between dose increments of 15 min, until reaching regular uterine dynamics (3–4 contractions/10 min) or maximum dose of 20 mU/min (120 mL/h).

The established indications for cesarean section during IOL are as follows: (1) failed IOL (FI): if after 12 h of regular uterine dynamics and IV oxytocin, with rupture of the amniotic membranes, it is not possible to establish an active phase of labor (in a patient with a previous cesarean section, this is 9 h); (2) non-progressive labor (NPL): when more than 4 h have elapsed in the active phase of labor, with ruptured membranes, and dilation has not progressed (in a patient with a previous cesarean section, this is 3 h); (3) cephalopelvic disproportion: in full dilation, regular contractions, and active pushing, the guiding point of the presentation does not go beyond the III plane of Hodge in women with epidural analgesia, 3 h in multiparous women, and 4 h in primiparous women (1 and a half hours in previous cesarean sections). In women without epidural analgesia, the time allowed is 2 h in multiparous women and 3 h in primiparous women (1 h in previous cesarean sections); (4) NICHD III CTG pattern not responding to measures to promote fetal oxygenation.

Fetal indications for IOL include the following: non-reassuring fetal heart rate (NRFHR), oligohydramnios, polyhydramnios, fetal growth restriction, small for gestational age, and macrosomia.

Maternal indications for IOL include the following: maternal diseases, such as gestational or pregestational diabetes, cholestasis, chronic hypertension or hypertensive diseases of pregnancy, poor obstetric history, and elective induction.

### 2.3. Statistical Analysis and Software Used

First, descriptive statistics were performed with absolute and relative frequencies for categorical variables and mean with standard deviation (SD) for quantitative variables.

We then performed a bivariate analysis to determine the sociodemographic and clinical differences between the group of women with a history of prior cesarean section and the group of women without prior cesarean section.

Next, bivariate and multivariate analyses were carried out between the existence of a previous cesarean section and the different obstetric and neonatal outcomes. For this, binary logistic regression or multiple linear regression was used depending on whether the result variable was categorical or quantitative in nature. Based on this, odds ratios (OR)/adjusted odds ratios (AOR) or mean differences (MD)/adjusted mean differences (AMD) were estimated with their respective 95% confidence intervals. All analyses were conducted using the program SPSS v24.0.

## 3. Results

**Figure 1:**
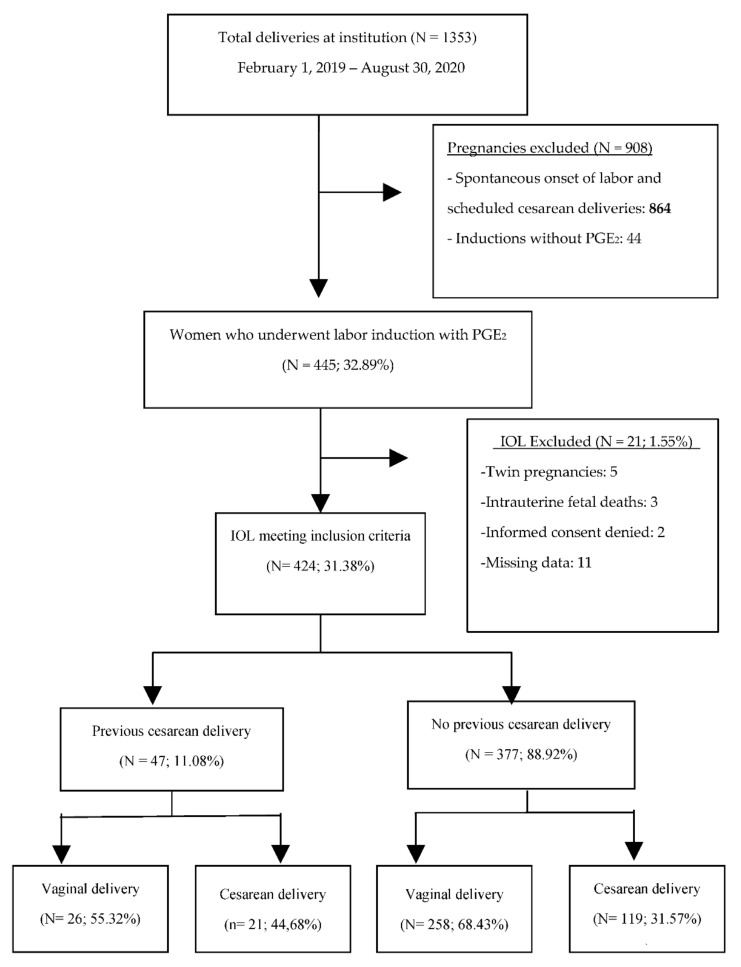
Flow chart of the selection process of the patients studied.

Of the total 1353 patients treated in the delivery unit of La Mancha Centro hospital between February 2019 and August 2020, 445 women underwent PGE_2_ (32.9%). Multiple gestations, non-cephalic presentations, antepartum fetal deaths, and those women who did not consent to participate in the study were excluded. Of the final 424 women included in the study, 47 women (11.1%) had a previous cesarean delivery, compared to 377 women without a previous cesarean section (88.9%). Figure 1 shows the flow chart of the selection process for the patients studied.

### 3.1. Characteristics of the Women Undergoing IOL according to History of Previous Cesarean Delivery

In the group of women with a previous cesarean section, the mean age was 34.21 (SD = 5.26) years, with a pre-pregnancy body mass index (BMI) of 29.81 (SD = 4.48) kg/m^2^. The mean cervical length (CL) was 34 (SD = 29.9) mm, with a Bishop score at the beginning of IOL of 2.08 (SD = 1.38), and 5.34 (SD = 2.54) at the time of admission to the delivery room. In the group of women with no previous cesarean section, the mean age was 32.75 (5.05) years, with a pre-pregnancy BMI of 30.06 (SD = 5.27) kg/m^2^. The mean CL was 27.7 (26.53) mm, with an initial Bishop score of 2.31 (SD = 1.55), and a score of 5.76 (SD = 2.47) after the cervical ripening process, at the time of admission to the delivery room. No statistically significant differences were observed when comparing the groups, except for the history of diabetes during pregnancy. Table 1 details the sociodemographic and obstetric characteristics of the pregnant women undergoing IOL.

### 3.2. Obstetric Outcomes according to History of Previous Cesarean Section

Among the pregnant women with a previous cesarean section, 21 had a cesarean delivery (44.7%), while, in the group of women without a previous cesarean section, 119 women (31.6%) had a cesarean delivery, with an AOR for cesarean delivery of 1.40 (95% CI: 0.68, 2.86). The response rate to cervical ripening was 55.32% (25/47), while, in the group without a previous cesarean section, it was 56.2% (212/377). No statistically significant differences were observed in the bivariate and multivariate analyses, in terms of the duration of the first and second stages of labor, presence of meconium, intrapartum fever, changes in the CTG, postpartum hemorrhage, uterine rupture, or admission to the ICU (*p* > 0.05). There were two cases of complete uterine rupture (4.3%), which were managed conservatively, in the group of patients with a prior cesarean section, and no cases of rupture in the group without a prior cesarean section. The obstetric results can be consulted in detail in Table 2.

### 3.3. Neonatal Morbidity

When analyzing neonatal outcomes and their relationship with IOL after a previous cesarean section, a greater probability for the need of type III–IV resuscitation was observed when performing the bivariate analysis (OR: 4.92; 95% CI: 1.38, 17.48), and a higher percentage of scores less than 7.20 (OR: 2.54; 95% CI: 1.08, 5.96) and less than 7.10 (OR: 17.66; 95% CI: 1.57, 199.0), in the group with a previous cesarean section. However, after performing the multivariate analysis, no statistically significant differences were observed in any of the neonatal variables studied (*p* > 0.05). Table 3 shows the neonatal results obtained.

The principal results that describe the study population are shown in Table 1. In terms of their family situation, 75.5% (117) of the patients were married and 16.1% (25) were widowed. The majority, 85.5% (133), of the patients lived with their families, and 4.5% (7) were institutionalized. The majority were retired, 73.5% (114), and 82.6% had primary level education.

## 4. Discussion

This comparative study was carried out to determine the relationship between having a history of a previous cesarean section, and important obstetric and neonatal results in IOL with PGE_2_. A total of 424 pregnant women were included, of which 11.1% presented a previous cesarean section. Although no statistically significant differences were observed when performing multivariate analysis, there were two cases of complete uterine rupture (4.3%) in the group of patients with a previous cesarean section. Additionally, in the bivariate analysis, the group with a previous cesarean had a greater probability of needing advanced neonatal resuscitation, and a higher percentage of umbilical artery pH values below 7.20 and 7.10.

In this sense, the main maternal risk that has been associated with IOL was uterine rupture. A recent meta-analysis of 69 studies on uterine rupture, in women undergoing IOL with PGE_2_, estimated a combined prevalence of five uterine ruptures per 1000 inductions of labor (95% CI: 2, 9) in patients with a history of a previous cesarean section. This risk increased to 11 per 1000 with the use of IV oxytocin [15]. However, the incidence of uterine rupture was only 0.7 women per 10,000 in IOL patients without a previous cesarean section. Therefore, it is a rare adverse event during the IOL process, and patients with a desire to attempt vaginal delivery after a previous cesarean section should be informed of this risk and offered the freedom of decision regarding initiating an induction process. Some authors, such as Guise et al., indicate that 370 elective cesarean sections are necessary to prevent one symptomatic uterine rupture [16]. In our study, we observed a uterine rupture rate of 4.3%, a figure that is much higher than that estimated globally by the meta-analysis by Chiossi et al. [15], and only surpassed by 2 of the 49 included studies [12,17]. In this sense, we believe that this high incidence may be due to random error and the small sample size used. We consider that, with a larger sample size, the incidence would probably be much lower than that observed.

Regarding this very relevant aspect, it is remarkable that only a small number of studies comparing IOL with PGE_2_ in women with a previous cesarean section vs. women with an intact uterus have been published in the literature, with the majority of these being retrospectives [18]. Among the studies identified, Locatelli et al. [19] observed a cesarean delivery rate of 29% in a cohort of 310 women with a previous cesarean section vs. 13.8% (*p* < 0.001) in a cohort of 5419 women without a previous cesarean section, as well as a rate of uterine rupture of 0.3% vs. 0.02% in the control group (*p* = 0.22). However, no significant differences were reported regarding the rest of the obstetric and neonatal variables analyzed (Apgar test scores at 5 min <7 or umbilical arterial PH <7). Similarly, MA Williams [20] evaluated the efficacy and safety of using 0.5 mg of PGE_2_ in an intracervical gel format, in a cohort of 117 women with a previous cesarean section vs. 354 nulliparous women. Compared with the control group, a higher cesarean delivery rate was obtained in women with a previous cesarean section (31.9% vs. 49.6%, respectively) (RR = 1.6, 95% CI 1.2–2.1). There were also no significant differences in maternal or fetal morbidity between the two groups.

Some authors have historically questioned the safety of PGE_2_ use in multiparous women with uterine scarring [21,22]. Haas et al. [23] conducted a retrospective study of 219 multiparous women (≥5 vaginal deliveries) with a prior cesarean section vs. 1376 multiparous women without a prior cesarean section, all of whom were undergoing IOL. Compared with the control group, a higher rate of cesarean section (6.84% vs. 3.4%, *p* < 0.001) and instrumental delivery (4.56% vs. 2%, *p* < 0.05) was observed in the study group. No significant differences were observed in the postpartum hemorrhage rate (0.91% vs. 0.90%, *p* = 0.2) or Apgar test scores at 5 min <7 (0.91% vs. 0.22%, *p* = 0.28) between both groups, and no cases of uterine rupture were reported either.

Therefore, it is difficult to fully answer the question of whether dinoprostone is a completely safe method in this population group, with so few comparative and retrospective studies. Therefore, the best approach to reliably determine the safety of this method in patients with uterine scarring would be to carry out a larger number of prospective studies, or high-quality randomized clinical trials, with adequate statistical power to allow firm conclusions to be drawn.

### Limitations and Strengths

Our study has various limitations to consider. First, the sample size of the patients with a history of a previous cesarean section, who underwent IOL, was small and insufficient to detect differences in severe, but infrequent, outcomes, such as uterine rupture, postpartum hemorrhage, admission to the ICU, or neonatal resuscitation III–IV. Hence, the data should be interpreted with caution. Another aspect to consider is that our results cannot be extrapolated to other hospitals in Spain, as the results of the cervical ripening process and IOL may be different in other centers, depending on the induction protocol and dose of oxytocin used.

After reviewing the literature on this method, we realized that there are no prospective studies evaluating the use of vaginal dinoprostone in patients with a previous cesarean section versus patients without a previous cesarean section. Despite not observing statistically significant differences, due to the sample size used, we did observe percentage differences in the obstetric and neonatal results obtained. We believe that this study could contribute to future systematic reviews focused on the use of vaginal dinoprostone as a method of IOL in patients with a previous cesarean section, which could provide greater clarity and robustness in the conclusions on the use of this method in this population group. In addition to its prospective nature, we established strict selection criteria to avoid confounding factors, and a single induction protocol was followed, with well-defined obstetric and neonatal variables.

## 5. Conclusions

Our data suggest that IOL with vaginal dinoprostone (PGE_2_), in pregnant women with a previous cesarean section, does not appear to be associated with worse obstetric or neonatal outcomes compared to IOL in pregnant women without a previous cesarean section, observing a response rate of 53.2% to cervical ripening with PGE_2,_ and a vaginal delivery rate of 55.32%. However, this induction method should be carried out with particular caution in this population group, using induction protocols and standardized IV doses of oxytocin.

## Figures and Tables

**Table 1:** Characteristics of the patients undergoing induction of labor (IOL) with vaginal dinoprostone according to previous history of cesarean section.

Variable	Previous Cesarean Delivery		*p* Value
	No	Yes	
Maternal characteristics			
Maternal age (years) *	32.75 (5.05)	34.21 (5.26)	0.064
Pregestational weight (kg) *	69.01 (14.56)	70.03 (13.99)	0.649
Antepartum weight (kg) *	80.33 (15.03)	80.01 (13.30)	0.888
Pregestational Body Mass Index (BMI) (kg/m^2^) *	30.06 (5.27)	29.81 (4.48)	0.756
Obstetrical characteristics			
Previous cesarean delivery			
Primiparity	245 (65)	35 (74.5)	0.196
Multiparity	132 (35)	12 (25.5)	
Preexisting or gestational diabetes			
No	342 (90.7)	43 (91.5)	0.049
Preexisting diabetes	3 (0.8)	1 (2.1)	
Gestational diabetes	32 (8.5)	3 (6.4)	
Hypertensive disease of pregnancy			
No	343 (91)	41 (87.2)	0.449
Chronic hypertension	8 (2.1)	0 (0)	
Gestational hypertension	14 (3.7)	4 (8.5)	
Preeclampsia	11 (2.9)	2 (4.3)	
Preeclampsia with severe features	1 (0.3)	0 (0)	
Intrauterine growth restriction (IUGR)			
No	357 (94.7)	47 (100)	0.106
Yes	20 (5.3)	0 (0)	
Obstetrical management			
Gestational age at birth (weeks)			
<37 + 0 days	13 (3.4)	2 (4.3)	0.890
≥37 + 0 days	364 (96.6)	45 (95.7)	
Cervical length prior to IOL, mm (CL) *	27.7 (26.53)	34 (29.19)	0.116
Prepartum amniotic fluid index (AFI)			
Normal	312 (82.8)	36 (76.6)	0.582
Oligoamnios	42 (11.1)	7 (14.9)	
Hydramnios	23 (6.1)	4 (8.5)	
Bishop score upon admission *	2.31 (1.55)	2.08 (1.38)	0.333
Bishop score upon admission to labor * room	5.76 (2.47)	5.34 (2.54)	0.264
Indication for induction			
Chronologically prolonged pregnancy	121 (32.1)	17 (36.2)	0.869
PROM	85 (22.5)	9 (19.1)	
Fetal	92 (24.4)	10 (21.3)	
Maternal	79 (21.0)	11 (23.4)	
Artificial rupture of membranes			
No	292 (77.5)	38 (80.9)	0.597
Yes	85 (22.5)	9 (19.1)	
Oxytocin use			
No	90 (24.5)	11 (23.4)	0.866
Yes	277 (75.5)	36 (76.6)	
Analgesia regional			
No	35 (9.3)	1 (2.1)	0.097
Yes	342 (90.7)	46 (97.9)	
Total duration of IOL (minutes) *	1236.38 (657.23)	1335.08 (623.75)	0.330
Birth weight (kg) *	3227.8 (498.3)	3199.3 (441.7)	0.709

* Mean (standard deviation).

**Table 2:** Obstetric results and their relationship to a history of previous cesarean section.

Variable	Previous Cesarean Delivery		Univariate Analysis		Multivariate Analysis **	
	No	Yes	OR/MD 95% CI	*p* Value	OR/MD 95% CI	*p* Value
Bishop score >6 after PGE_2_						
No	165 (43.8)	22 (46.8)	0.88 (0.48, 1.62)	0.692	1.14 (0.57, 2.31)	0.706
Yes	212 (56.2)	25 (53.2)				
Duration of dilatation (min) *	359.93 (229.82)	362.19 (216.52)	−2.26 (−72.80, 68.28)	0.950	−19.40 (−76.76, 37.96)	0.506
Duration third stage of labor (min) *	94.22 (81.36)	94.08 (60.81)	0.15 (−32.82, 33.12)	0.993	7.09 (−19.80, 34.17)	0.606
Type of delivery						
Vaginal delivery	258 (68.4)	26 (55.3)	1.75 (0.95, 3.24)	0.074	1.40 (0.68, 2.86)	0.358
Cesarean delivery	119 (31.6)	21 (44.7)				
Meconium						
No	324 (85.9)	40 (85.1)	1.07 (0.45, 2.51)	0.877	1.27 (0.52, 3.11)	0.591
Yes	53 (14.1)	7 (14.9)				
Intrapartum fever						
No	357 (94.9)	44 (93.6)	0.78 (0.22, 2.74)	0.699	0.83 (0.22, 3.08)	0.784
Yes	19 (5.1)	3 (6.4)				
CTG ^1^: NICHD ^2^ 2						
No	253 (70.3)	28 (65.1)	1.27 (0.65, 2.47)	0.487	1.07 (0.54, 2.13)	0.844
Yes	107 (29.7)	15 (34.9)				
CTG ^1^: NICHD ^2^ 3						
No	253 (70.3)	28 (65.1)	2.13 (0.67, 6.76)	0.201	3.03 (0.87, 10.54)	0.082
Yes	107 (29.7)	15 (34.9)				
Postpartum hemorrhage ^a^						
No	349 (93.3)	42 (89.4)	1.66 (0.60, 4.57)	0.325	1.62 (0.56, 4.65)	0.371
Yes	25 (6.7)	5 (10.6)				
Uterine rupture ^b^						
No	375 (100)	45 (95.7)				
Yes	0 (0)	2 (4.3)	NC	0.993	NC	0.979
Blood loss > 3.5 (1)						
No	351 (94.4)	47 (100)	NC	0.997	NC	0.997
Yes	21 (5.6)	0 (0)				
ICU ^3^ admission						
No	372 (99.5)	47 (100)	0 (NC)	0.998	0 (NC)	0.997
Yes	2 (0.5)	0 (0)				
Need for transfusion						
No	362 (97.3)	46 (97.9)	0.78 (0.09, 6.29)	0.821	0.67 (0.07, 6.06)	0.721
Yes	10 (2.7)	1 (2.1)				

OR: odds ratio; MD: mean difference; CI: confidence interval. ^a^ Postpartum hemorrhage defined as more bleeding than expected with signs and symptoms of hypovolemia, for which the gynecologist had to initiate uterotonic drugs. ^b^ Uterine rupture: complete rupture of all uterine layers, including the serosal layer. NC: not calculated.^1^ CTG: cardiotocography. ^2^ NICHD: CTG classification based on the system proposed by the National Institute of Child Health and Human Development.^3^ ICU: intensive care unit. * Mean (standard deviation). ** Multivariate analysis adjusted for maternal age, hypertension, diabetes, BMI, parity, regional analgesia use, Bishop score on admission, use of oxytocin, and neonatal weight.

**Table 3:** Neonatal results and their relation to a history of previous cesarean section.

Variable	Previous Cesarean		Univariate Analysis		Multivariate Analysis **	
	No	Yes	OR 95% CI	*p* Value	OR 95% CI	*p* Value
APGAR < 7 at 1 min						
No	367 (97.3)	45 (95.7)	1.63 (0.35, 7.68)	0.536	1.25 (0.23, 6.76)	0.799
Yes	10 (2.7)	2 (4.3)				
APGAR < 7 at 5 min						
No	376 (99.7)	46 (97.9)	8.17 (0.50, 132.90)	0.140	NC	0.999
Yes	1 (0.3)	1 (2.1)				
Admission to neonatal unit						
No	337 (89.4)	41 (87.2)	0.81 (0.32, 2.03)	0.655	0.90 (0.32, 2.53)	0.844
Yes	40 (10.6)	6 (12.8)				
REA III-IV						
No	370 (98.1)	43 (91.5)	4.92 (1.38, 17.48)	0.014	3.91 (0.83, 18.36)	0.084
Yes	7 (1.9)	4 (8.5)				
U. artery pH < 7.20 at birth						
No	333 (91.7)	35 (81.4)	2.54 (1.08, 5.96)	0.033	2.32 (0.91, 5.89)	0.077
Yes	30 (8.3)	8 (18.6)				
U. artery pH < 7.10 at birth						
No	362 (99.7)	41 (95.3)	17.66 (1.57, 199.00)	0.020	NC	0.892
Yes	1 (0.3)	2 (4.7)				

OR: odds ratio; CI: confidence interval. APGAR: scoring system for the newborn (appearance, pulse, grimace, activity, respiration). REA: level of resuscitation required at birth. U. artery: umbilical artery. ** Multivariate analysis adjusted for prematurity, newborn weight, CTG classification, ruptured membranes, intrapartum fever, meconium, IUGR, hypertension, and diabetes.

## Data Availability

The data sets generated and/or analyzed during the current study are available from the corresponding author on reasonable request.
